# Supplementary material for: Don’t put all social network sites in one basket: Facebook, Instagram, Twitter, TikTok, and their relations with well-being during the COVID-19 pandemic
Source: PLoS One. 2021 Mar 11;16(3):e0248384. doi: 10.1371/journal.pone.0248384 (PMC7951844; doi:10.1371/journal.pone.0248384)
Supplement: Attachment — Submitted filename: Response to Reviewers.docx [file pone.0248384.s001.docx]

**Response to the Editor and the Reviewers**

We are re-submitting in PLOS One our paper "Don’t put all social network sites in one basket: Facebook, Instagram, Twitter, TikTok, and their relations with well-being during the COVID-19 pandemic.".

We thank the editor and the reviewers for their time spent carefully reviewing our manuscript, and for their valuable comments. We made sure that each one of the reviewer’s comments has been addressed carefully; we believe that the manuscript has been really improved.

Here are the major revisions made to the manuscript:

- Regarding the unbalance-sample, dependent variables were controlled for gender and age. This change did not affect the models for Facebook, Instagram and TikTok, but led to substantial revisions in the Twitter’s model. We think that this contribution enhances the scope of our discussion.
- The definition of what is meant by a passive and an active usage of social network sites had been more detailed.
- As requested, we made our date available on an online repository; the DOI necessary to access our data is (*anonymized link for blind peer review*):	<https://osf.io/s5mjx/?view_only=b852a4a3eb884b8bb11b83256bee0161>.
  We also made sure that the manuscript meets PLOS ONE's style requirements.

Minor revisions, if not explained, are applied to the manuscript, mostly for correcting typing errors, and for minor modification along with revisions explained in this document.

Below are detailed responses to all the reviewer’s comments. The latter are shown in black and our responses in blue.

Please let us know if you still have any questions or concerns about the manuscript. We will be happy to address them.

Sincerely,

The authors of paper PONE-D-20-27590.

**Page**

[Reviewer #1	2](#_Toc57391871)

[Reviewer #2	4](#_Toc57391872)

[Reviewer #3	8](#_Toc57391873)

# Reviewer #1

This article deals with an interesting question: the relationship between social network usage and well-being during the COVID-19 pandemics. The article expands the horizon of the review made by Verduyn et al. (2017), by providing an analysis through multiple platforms, based on structural equation modeling. The research design is similar to the one described in Verduyn et al. (2017), with the same two mediators: social support and upward social comparison.
The procedure is correct, and the contribution of the work comes from the results it presents, which might help understand human interactions through social networks. From the methodological point of view, I think the work is not innovative. However, the procedure is sound and the literature review is also correct.

As I flaw, I found the data quite biased towards the female population (line 139): 77% females vs. 23% males, which the authors recognize as a limitation. I wonder if it is also biased towards certain socio-economic segments and age segments, as the cohort was obtained from an academic mailing list. Though this is common in many research works in this area, I think that this type of limitations should be briefly discussed.

We totally agree with the reviewer’ comment; we have further elaborated our discussion to better consider this kind of limitation: “**Since participants were recruited via academic mailing lists from social science, the sample is quite biased towards academic people, as well as women (there are a majority of women in social science). This kind of limitation is common in research about social network sites, but we could suspect that this unbalance-sample limits the generalization of the results. Likewise, the small number of participants having a TikTok account, and the fact that all participants were Francophone, highlight the need to replicate the study in other populations.**” (page 17, lines 396-402).

In addition, we have also redone the analyses to control for gender and age: “**For each model, dependent variables were controlled for age and gender.**” (results section, page 10, line 235). Results did not changed for Facebook, Instagram and TikTok, but did for Twitter: “**In other words, results showed that actively using Twitter was associated with more social support, and that using passively Twitter was associated with less upward social comparison. In addition, social support mediated the relation between using actively Twitter and satisfaction with life, and social comparison mediated the relation between passively using Twitter and negative affect.**” (results section, page 14, lines 312-316). The discussion section was therefore modified in light of these new findings (see discussion section, page 16, lines 358-376).

In general lines, and despite its limitations, I think that the work can be considered for publication in PLOS ONE.

Minor details:

- This phrase seems contradictory (line 89) "Recently, Chae (21) showed a negative relationship between the use of Twitter, Instagram, LinkedIn and relative well-being through social comparison. Specifically, Instagram and LinkedIn enhanced social comparison, whereas Twitter decreased social comparison." I guess that the first sentence should be neutral: "Recently, Chae (21) showed a negative relationship between the use of Twitter, Instagram, LinkedIn and relative well-being through social comparison. Specifically, ....". Otherwise, it would be interpreted that there it showed a negative relationship between the use of Twitter and relative well-being through social comparison, and I think it was not the case. Please check.

We have revised the text to address your concerns and hope that it is now clearer: “**Recently, Chae has also examined the relationships between various platforms and well-being through social comparison. As expected, social comparison was negatively associated with well-being; but while Instagram and LinkedIn enhanced social comparison, Twitter decreased it. Surprisingly, Facebook use was not related to well-being.**” (pages 4-5, lines 95-99).

- Line 213: "khi-deux" should read "chi-square".

We agree and have updated the sentence (page 11, line 239).

Finally, I remark that the data used in this research has not been made available with the submission. I understand that the authors will make it public after acceptance, according to the PLOS ONE policy on Data Availability (<http://journals.plos.org/plosone/s/data-availability>). Data availability is mandatory, and should be checked before publication.

We thank the reviewer for pointing out our forgetfulness. We have made the necessary change: “**Measures reported in the present study are part of a larger questionnaire; all data are available in OSF (Open Science Framework) at: https://osf.io/s5mjx/?view_only=b852a4a3eb884b8bb11b83256bee0161 (*anonymous link for blind peer-review)***” (page 7, lines 160-163).

Finally, we specifically thank the reviewer for the positive appreciation his/her gave to our manuscript, and for the time his/her spend reading our work. We hope our clarifications made the manuscript sounder.

# Reviewer #2

In this paper the authors present a study concerning possible correlations of the active/passive usage of social networks and medias and well-being, positive effects and negative effects. The paper seems overall well written, although some typos were found, but there are a number of very important weaknesses listed below.

1) All the key concepts of this paper are not well defined and are extremely vague. I did not find any definition of "well-being", for instance. Is it related to "being healthy"? In this case probably the model is not complex enough to capture the concept of "well-being" because it lacks other important aspects. The same goes for "Satisfaction with life", Positive affects", and "Negative affects". The clarification of these concepts would make the paper clear/easy to understand and technically sound.

We have further elaborated the paragraph which defined well-being in the part “1.1 Definition of key concepts”. We have changed “On the other side, subjective well-being comprises three distinct factors: the presence of positive affects, the absence of negative affects, and a sense of satisfaction with life (8). Typically, positive and negative affects reflect the affective level of well-being, and satisfaction with life corresponds to the cognitive level.” to “**On the other side, the term well-being refers, in this study, to the subjective part of well-being. Instead of relying on physical and material resources, subjective well-being can be understood as “people’s overall evaluations of their lives and their emotional experiences.” (8 p. 87). Hence, subjective well-being is a multidimensionality construct, and each component needs to be assessed individually. Typically, subjective well-being comprises at least three components: a sense of satisfaction with life, the presence of positive affect, and the absence of negative affect (8). The satisfaction with life allows to capture how people evaluate their lives (i.e., cognitive level of subjective well-being). Likewise, positive and negative affect reflect what feelings people experience in their lives (i.e., affective level of subjective well-being).**” (pages 3-4, lines 66-74). We hope this is a sufficient clarification.

Diener, E., Heintzelman, S. J., Kushlev, K., Tay, L., Wirtz, D., Lutes, L. D., & Oishi, S. (2017). Findings all psychologists should know from the new science on subjective well-being. *Canadian Psychology/Psychologie Canadienne, 58*(2), 87–104. <https://doi.org/10.1037/cap0000063>

2) In Section 2 there is a discussion concerning the 4 sns used for the analyses. A part of the discussion can be easily summarised considering the fact that Facebook is a Social Network (build a network of known people), while the other three are Social Media (media sharing platforms). I agree that the study should be carried considering all the platforms separately, but I somewhat expect some similarities between social media platforms, especially between Instagram and TikTok.

We agree that it would have been useful to demonstrate some similarities between Instagram and TikTok, which are both based on image. Regarding the term “social network site”, Instagram, TikTok and Twitter are fully in line with the definition of Ellison and boys (2013): users have a uniquely identifiable profiles, they can articulate public connections, and they can produce content and interact/consume the content of their connections. However, we agree that connections on Facebook are mainly composed of known people. That is the reason why we have highlighted the motivations to use social network sites: Facebook is used to interact with people, contrary to TikTok and Twitter for example.

Ellison, N. B., & boyd, D. (2013). Sociality through social network sites. In W. H. Dutton (Ed.), *The Oxford handbook of Internet studies* (pp. 151–172). Oxford University Press.

3) The dataset does not seem to be relevant for the study.
- It is made of only 793 people
- The number of women is much higher. In many datasets it was shown that usually the number of females and males on sns are similar or only slightly unbalanced. But not 75% women and 25% men.
- They are all francophone, thus probably living in a specific geographic region
- Participants were recruited using academic mailing lists, thus they are probably all academic people.
- The number of people using Twitter or TikTok seems very low.
The low number of people and all the other very specific features makes me think that probably there is a very strong bias in the dataset.

We agree with the reviewer that our sample is not a representative of the population. However, our study does not convey this ambition. We believe that our results open the reflection on the specificities of each social network sites, and we agree that further studies will be necessary.

These limitations led us to add supplementary information in the paper:

- “**Regarding the highest degree completed, one person had no primary education, 253 had a high school degree, 285 had a university short cycle degree (two or three years), 207 had a university long cycle degree (four or five years), and 47 had a doctorate.**” (method section, page 7, lines 151-153)

- “**Participants were recruited through academic mailing lists from social science, which explains the large proportion of women and academic people in the sample.**” (method section, page 7, lines 156-158)

- “**For each model, dependent variables were controlled for age and gender.**” (results section, page 10, line 235)

- “**Since participants were recruited via academic mailing lists from social science, the sample is quite biased towards academic people, as well as women (there are a majority of women in social science). This kind of limitation is common in research about social network sites, but we could suspect that this unbalance-sample limits the generalization of the results. Likewise, the small number of participants having a TikTok account, and the fact that all participants were Francophone, highlight the need to replicate the study in other populations.**” (discussion section, page 17, lines 396-402)

4) The authors should spend more effort in motivating the methodology. Here are some questions:
- line 154: Why did you use a 7-point scale?

Most studies on social network sites use these kinds of scale to measure the frequency of use (for example, Kittinger, Correia, & Irons, 2012). Besides, to ensure consistency and not confusing participants, we have standardized the point questions to seven for all measures in the questionnaire. Our objective was therefore to facilitate the administration of the questionnaire.

Kittinger, R., Correia, C. J., & Irons, J. G. (2012). Relationship between Facebook use and problematic Internet use among college students. *Cyberpsychology, Behavior, and Social Networking, 15*(6), 324-327.

- lines 159-160: does that mean that a user can be both very active and very passive at the same time? And what does "very actively" means? Once per day is "very active"?

That is exactly the point: users can be both very active and very passive at the same time (Gerson, Plagnol, & Corr, 2017). Indeed, since social network sites have different features and activities, a user can spend one hour to scrolling through one’s news feed, while spending another hour to try photo filters. Participants had also a description of what is considered as a passive and an active usage.

We apologize for this lack of information. We have updated the measures section to integrate these additional clarifications “**Active use was defined as “posting and commenting on [Facebook][Instagram][Twitter][TikTok], for example: post content on your profile, react to posts and comments from other users, etc.”, while passive use as “browsing [Facebook][Instagram][Twitter][TikTok], for example: scrolling through your news feed, looking at other users' profiles, etc.”. In other words, we consider that users can have both an active and a passive usage of SNSs; they can spend most of their time scrolling their news feed, but they can also send messages throughout the day.**” (page 8, lines 173-179).

Gerson, J., Plagnol, A. C., & Corr, P. J. (2017). Passive and Active Facebook Use Measure (PAUM): Validation and relationship to the Reinforcement Sensitivity Theory. *Personality and Individual Differences, 117*, 81–90. <https://doi.org/10.1016/j.paid.2017.05.034>

- line 172: you use Likert scale extensively in your paper. A relevant citation would increase the quality of the paper. Additionally, why did you use that scale? are there alternatives? Why is Likert the best choice in this scenario?

We chose Likert scales because they were used in the Satisfaction with Life Scale (Diener, Emmons, Larsen, & Griffin, 1985) and the motivations scale to use SNSs (Cheung, Chiu, & Lee, 2011), and we did not want that participants manage various measurement scales. In addition, this technique is easy to understand, commonly used in social science research and highly reliable (Likert, 1932).

Cheung, C. M., Chiu, P.-Y., & Lee, M. K. (2011). Online social networks: Why do students use facebook? *Computers in Human Behavior, 27*(4), 1337–1343. https://doi.org/10.1016/j.chb.2010.07.028

Diener, E., Emmons, R. A., Larsen, R. J., & Griffin, S. (1985). The Satisfaction With Life Scale. *Journal of Personality Assessment, 49*(1), 71–75. <https://doi.org/10.1207/s15327752jpa4901_13>

5) I would spend more effort also in the reorganisation of the contents in the paper: sometimes results (like the McDonald's omega) is shown in the framework presentation section, and part of the framework (lines 210-218) is presented in the result section. You should separate better the framework from the results to help readability.

We agree with reviewer that the presentation of results can be sometimes difficult to read. However, we have followed the Journal Article Reporting Standards (JARS) of the American Psychological Association (APA): the internal consistency coefficients (like the McDonald's omega) have to be presented in the method section, while the details of the models estimated (the method used for SEM, the fit indices chosen, etc.) have to be presented in the results section (Appelbaum, Cooper, Kline, Mayo-Wilson, Nezu, & Rao, 2018).

Appelbaum, M., Cooper, H., Kline, R. B., Mayo-Wilson, E., Nezu, A. M., & Rao, S. M. (2018). Journal article reporting standards for quantitative research in psychology: The APA Publications and Communications Board task force report. *American Psychologist, 73*(1), 3-25. http://dx.doi.org/10.1037/amp0000191

typos:
line 35: "Analyzes employed", plural of "analysis" is"analyses"
lines 60-64: probably there is some problems with the indentation of the text here

We took care of the typo “analyses” as indicated by the reviewer (page 2, line 34).

Regarding the indentation of the text (page 3, lines 61-65), we followed the norm of the APA for block quotation (40 words or more): not using quotation marks, and starting “a block quotation on a new line and indent the whole block 0.5 in. from the left margin” (<https://apastyle.apa.org/style-grammar-guidelines/citations/quotations>).

Finally, we thank the reviewer for his/her careful reading of the manuscript. We believe that his/her comments helped us to better communicate our work, extending therefore the scope of this study.

# Reviewer #3

The manuscript in question approaches an important problem of how different types of social networks affect subjective well-being of users.

The authors design a thorough survey that includes assessment of such measures as motivation, social support, upward social comparison etc.
Then they use a well established framework of structural equation modeling (SEM) to evaluate direct and indirect effects of active and passive use of social networks on well-being comprised of satisfaction with life, positive affect and negative affect.


1) The paper would benefit from exploratory data analysis: how do the distribution of measures look, what are correlations between them? It is an important first step that can serve as a sanity check when using SEM.

We thank the reviewer for pointing this out. We have made these exploratory data analyses; they are available online in OSF. We have added a sentence in the manuscript for interested readers: “**A statistical summary of all the variables (i.e., descriptive statistics, and correlation table) is available in OSF at:** [**https://osf.io/s5mjx/?view_only=b852a4a3eb884b8bb11b83256bee0161**](https://osf.io/s5mjx/?view_only=b852a4a3eb884b8bb11b83256bee0161) **(*for blind peer review, this link leads to the general project on OSF, but not directly to the statistical summary document. You can find it in the "Data Appendix" folder.***” (page 10, lines 217-221).

2)
a) Two genders are well represented in this study. It is clear that gender might be an important factor in determining how well-being is derived from the use of social networks, so it should included during modeling.
At the very least it would be interesting to compare the distributions of measures by gender.

We agree with the reviewer’ comment and think that we should have controlled for gender, but also age, which are known to affect well-being. We have therefore redone the analyses: “**For each model, dependent variables were controlled for age and gender.**” (page 10, line 235). Our findings did not change for Facebook, Instagram and TikTok, but did for Twitter: “**In other words, results showed that actively using Twitter was associated with more social support, and that using passively Twitter was associated with less upward social comparison. In addition, social support mediated the relation between using actively Twitter and satisfaction with life, and social comparison mediated the relation between passively using Twitter and negative affect.**” (results section, page 14, lines 312-316). In the discussion section, we discussed these findings in light of the literature: “**In other words, our results are fully consistent with those of Chae [21] and demonstrate that, rather than an absence of relation [20], both active and passive usage of Twitter can be positively related to well-being. Which might seem surprising - the negative association between passive usage of Twitter and upward social comparison – can find an explanation in the social context of Twitter. Indeed, previous studies have shown that negative messages are shared faster on Twitter [40] and that popular events on Twitter are associated with negative emotions [41]. Recently, Waterloo, Baumgartner, Peter and Valkenburg [42] showed than negative emotions are perceived as more appropriate on Facebook and Twitter, compared to Instagram. Hence, it is plausible that Twitter’s users scrolling through their Twitter news feed, and seeing constant bad news from their followers, are more inclined to compare their situation with what they consider to be worse (i.e. downward social comparison), rather than better off (i.e. upward social comparison).**” (page 16, line 358-374).

b) The gender ratio is imbalanced. Why is it the case? How does it compare to the gender ratio of the recipients of the survey invitation? Does it create a bias? Do those who are not likely to participate in surveys use social networks in the same way? Well, given the gender imbalance of this survey and if there are significant gender differences in SN use, we can hypothesize that those who haven't participated have the inverse gender ratio and so their average motivation and modus operandi might be very different.

The gender ratio is imbalanced because participants were recruited through academic mailing lists from social science, which are mainly composed of women. Therefore, we do not think that people who have not participated in the study have the inverse gender ratio, but rather that the participants reached through the data collection were mainly composed of women. We apologize for this lack of information.

We have adjusted the paper in consequence:

- “**Participants were recruited through academic mailing lists from social science, which explains the large proportion of women and academic people in the sample**.” (method section, page 7, lines 156-158)

- “**Since participants were recruited via academic mailing lists from social science, the sample is quite biased towards academic people, as well as women (there are a majority of women in social science). This kind of limitation is common in research about social network sites, but we could suspect that this unbalance-sample limits the generalization of the results.**” (page 17, lines 396-400)

3) It appears that the measures were deduced for users who potentially use a mix of social networks. So if someone uses both Facebook and Instagram how do we estimate the fraction that contributes to his motivation for each network?
It seems like a good model should include the use of all networks simultaneously.

We appreciate the reviewer’s insightful suggestion and agree that it would be useful to include the use of all networks simultaneously; however, such an analysis would require a larger group of participants regarding the number of paths. In addition, in view of the few participants having a Twitter and TikTok accounts, we think that the results will be biased. Nevertheless, we recognize this limitation should be mentioned in the paper, so we added the following sentence: “**Thirdly, as noted by an anonymous reviewer, we think that another good way of assessing our hypotheses would have been to test a model including all social network simultaneously. However, we think that this kind of modeling requires much more participants to draw valid conclusions.**” (discussion section, page 18, lines 407-410).

4) It would be nice to see a discussion of the impact of the survey invitation being circulated in academic mailing lists and the bias it potentially introduces.

To highlight the specificity of our data collection through academic mailing lists, we first gave more information about participants in the method section: “**Regarding the highest degree completed, one person had no primary education, 253 had a high school degree, 285 had a university short cycle degree (two or three years), 207 had a university long cycle degree (four or five years) and 47 had a doctorate.”** (page 7, lines 151-153), and we also made changes in the discussion section: “**Since participants were recruited via academic mailing lists, the sample is quite biased towards academic people, as well as women (there are a majority of women in social science). This kind of limitation is common in research about social network sites, but we could suspect that this unbalance-sample limits the generalization of the results.**” (page 17, lines 396-400).

5) Active and passive SN use are treated independently, if I understand correctly. But this is an assumption, and at the very least it should be discussed. It would be interesting to see if the three groups of those who are using actively, passively and both actively and passively have similar distributions of measures.

Active and passive usage are treated independently based on the assumption that user can have, at the same time, a passive and an active usage of social network sites (Gerson, Plagnol, & Corr, 2017). We agree with the reviewer that we should have discuss this assumption in the paper. We have therefore added more information in the measure section: “**Active use was defined as “posting and commenting on [Facebook][Instagram][Twitter][TikTok], for example: post content on your profile, react to posts and comments from other users, etc.”, while passive use as “browsing [Facebook][Instagram][Twitter][TikTok], for example: scrolling through your news feed, looking at other users' profiles, etc.”. In other words, we consider that users can have both an active and a passive usage of SNSs; they can spend most of their time scrolling their news feed, but they can also send messages throughout the day.**” (page 8, lines 173-179).

Gerson, J., Plagnol, A. C., & Corr, P. J. (2017). Passive and Active Facebook Use Measure (PAUM): Validation and relationship to the Reinforcement Sensitivity Theory. *Personality and Individual Differences, 117*, 81–90. <https://doi.org/10.1016/j.paid.2017.05.034>

Regarding the comparison of active and passive users, we distinguished four profiles of users: having a high passive and low active usages, having a high passive and a high active usages, having a low passive and a low active usages, and having a low passive and a high active usages. To do so, we used the Median Split method; the values below the variables’ median for passive and active usage have been put in the category “Low”, and those above in the category “High” (for more details, see the codebook in the OSF project).

Below are descriptive statistics for well-being based on these profiles (table 1, table 2, table 3, table 4). These tables show that the distributions of the measures are not similar between each profile. In addition, for each SNS, participants having a low passive usage and a high active usage were more satisfied with their lives compared to other profiles users.

Table 1. Descriptive statistics of well-being’s components according to Facebook users’ profiles.

| Facebook User profile | **Satisfaction with life** | | | **Positive affect** | | **Negative affect** | | *Total* |  |
| --- | --- | --- | --- | --- | --- | --- | --- | --- | --- |
|  | *Mean* | *SD* | | *Mean* | *SD* | *Mean* | *SD* |  |  |
| High Passive, Low Active | 4.402 | 1.490 | | 3.425 | 1.420 | 3.288 | 1.388 | *161* |  |
| High Passive, High Active | 4.483 | 1.401 | | 3.624 | 1.354 | 3.284 | 1.464 | *266* |  |
| Low Passive, Low Active | 3.912 | 1.326 | | 3.330 | 1.327 | 2.819 | 1.374 | *50* |  |
| Low Passive, High Active | 4.650 | 1.337 | | 3.931 | 1.448 | 3.119 | 1.633 | *226* |  |
|  |  |  |  |  |  |  |  |  |  |

Table 2. Descriptive statistics of well-being’s components according to Instagram users’ profiles.

| Instagram User profile | **Satisfaction with life** | | | **Positive affect** | | **Negative affect** | | *Total* |  |
| --- | --- | --- | --- | --- | --- | --- | --- | --- | --- |
|  | *Mean* | *SD* | | *Mean* | *SD* | *Mean* | *SD* |  |  |
| High Passive, Low Active | 4.453 | 1.412 | | 3.608 | 1.482 | 3.123 | 1.494 | *189* |  |
| High Passive, High Active | 4.224 | 1.336 | | 3.475 | 1.291 | 3.624 | 1.459 | *118* |  |
| Low Passive, Low Active | 4.407 | 1.720 | | 3.807 | 1.517 | 3.094 | 1.597 | *57* |  |
| Low Passive, High Active | 4.416 | 1.348 | | 3.493 | 1.326 | 3.461 | 1.512 | *138* |  |
|  |  |  |  |  |  |  |  |  |  |

Table 3. Descriptive statistics of well-being’s components according to Twitter users’ profiles.

| Twitter User profile | **Satisfaction with life** | | | **Positive affect** | | **Negative affect** | | *Total* |  |
| --- | --- | --- | --- | --- | --- | --- | --- | --- | --- |
|  | *Mean* | *SD* | | *Mean* | *SD* | *Mean* | *SD* |  |  |
| High Passive, Low Active | 4.388 | 1.416 | | 3.629 | 1.511 | 3.429 | 1.510 | *101* |  |
| High Passive, High Active | 3.949 | 1.321 | | 3.342 | 1.420 | 3.443 | 1.560 | *79* |  |
| Low Passive, Low Active | 4.104 | 1.459 | | 3.481 | 1.362 | 3.468 | 1.520 | *27* |  |
| Low Passive, High Active | 4.101 | 1.262 | | 3.398 | 1.305 | 3.617 | 1.426 | *93* |  |
|  |  |  |  |  |  |  |  |  |  |

Table 4. Descriptive statistics of well-being’s components according to TikTok users’ profiles.

| TikTok User profile | **Satisfaction with life** | | **Positive affect** | | **Negative affect** | | *Total* |  |
| --- | --- | --- | --- | --- | --- | --- | --- | --- |
|  | *Mean* | *SD* | *Mean* | *SD* | *Mean* | *SD* |  |  |
| High Passive, Low Active | 4.168 | 1.492 | 3.280 | 1.242 | 3.900 | 1.403 | *79* |  |
| High Passive, High Active | 4.440 | 1.037 | 3.467 | 1.217 | 3.687 | 1.592 | *20* |  |
| Low Passive, Low Active | 4.243 | 1.243 | 3.071 | 1.492 | 3.309 | 1.616 | *12* |  |
| Low Passive, High Active | 4.352 | 1.364 | 3.274 | 1.241 | 3.499 | 1.376 | *10* |  |

I conclude that the manuscript is an analysis of the effect social networks on personal well-being based of a large and feature-rich dataset. However, the dataset analysis is incomplete.
This manuscript requires a major revision. Once all the points raised in this review are addressed it can be published in PLOS.

Finally, we thank the reviewer for his/her sound comments, and specifically for the precious time he/she spent on our scientific method. We are very grateful for the opportunity to have such kind of advice.
