# Supplementary material for: Don’t put all social network sites in one basket: Facebook, Instagram, Twitter, TikTok, and their relations with well-being during the COVID-19 pandemic
Source: PLoS One. 2021 Mar 11;16(3):e0248384. doi: 10.1371/journal.pone.0248384 (PMC7951844; doi:10.1371/journal.pone.0248384)
Supplement: Attachment — Submitted filename: Response to Reviewers.docx [file pone.0248384.s002.docx]

**Response to the Editor and the Reviewers**

We are re-submitting in *PLOS One* our paper "Don’t put all social network sites in one basket: Facebook, Instagram, Twitter, TikTok, and their relations with well-being during the COVID-19 pandemic.".

We thank again the editor and the reviewers for the time they spent reviewing our manuscript and for their positive remarks. We made sure that each one of their concerns has been addressed carefully.

Below are detailed responses to all the reviewer’s comments. The latter are shown in black and our responses in blue.

Please let us know if you still have any suggestions about the manuscript. We will be happy to address them.

Sincerely,

The authors of paper PONE-D-20-27590.

**Page**

[Reviewer #1	2](#_Toc61000487)

[Reviewer #2:	3](#_Toc61000488)

[Reviewer #3:	5](#_Toc61000489)

# Reviewer #1

The authors have addressed all the remarks and the quality of the manuscript has been improved. In particular, they have correctly discussed the limitations, clarified some relevant definitions for their work, and made their dataset available.

I consider that the manuscript can be accepted for publication in PLOS ONE.

Minor note: I observe that the authors have changed the subsection title "Measures" into "Materials" on line 164. I think that "Measures" might be more appropriate and standard in the field.

We thank the reviewer for the time he/she spent reading and revising our manuscript, but also for considering that it can be accepted for publication in PLOS One. We changed the subsection title “Measures” when we were trying to meet the PLOS ONE's style requirements, but we agree with the reviewer that “Measures” is more suitable than “Materials”. We therefore made the necessary change (page 7, line 168).

# Reviewer #2:

Authors put an extraordinary effort to revise and improve the paper. I have just a few follow-up points:

We thank again the reviewer for his/her positive remarks, and for his/her valuable comments. Below are our detailed responses.

- line 33: you claim that 1008 people took the test, but on line 146 you claim that 1004 people took the test. Please, put the correct number in the paper.

We thank the reviewer for pointing this out! 1004 people participated in the study (the original data file is available at OSF: <https://osf.io/s5mjx/?view_only=b852a4a3eb884b8bb11b83256bee0161>). We have therefore updated the abstract section (page 2, line 33).

- lines 170-179: now it's much more clearer what "actively" and "passively means", but I still wonder whether it was a good idea to keep these two factors separated. In this way one can be "non active" and "non passive" at the same time which does not make much sense (or am I still missing something?). Additionally, if I got it right, they are mutually exclusive activities: if I am scrolling through posts, I am not creating posts at the very same time. I may spend equal time in active and passive behaviour, and that's why I think that a single indicator is better here. Can you please motivate in the paper why you needed two different indicators? You also asked the participants their "overall SNS use", but I don't see it used in the paper, why?

We thank the reviewer for his/her suggestion. We agree that our choice to keep these two factors separated requires a lengthy justification in the manuscript. On one hand, we have motivated our choice in the introduction section “**Gerson, Plagnol and Corr [13] pointed out how these usages match with specific SNS activities, demonstrating they reflect related, but separate constructs.**” (page 4, lines 84-85), and the method section “**To be consistent with the literature [13], we choose to measure passive and active usage as separate constructs. This means that users can have both an active and a passive usage; they can spend most of their time scrolling their news feed, but they can also send messages throughout the day.**” (page 8, lines 174-177). On the other hand, we discussed it as a limitation in the discussion section: “**Second, to avoid demotivating respondents, we have limited the questionnaire’s length. Consequently, passive and active SNS usages have been measured with one item. Although they are considered as separated constructs in the literature [13], we may suspect that the use of single items has increased their association. Future studies should therefore assess specific activities on each SNS.**” (page 17, lines 405-409). Regarding the overall SNS use, the literature highlights how the studies focusing on an overall social network sites use led to contradictory results. We therefore considered that the passive and the active usage are the most informative and the most relevant for our research question. However, we agree that these overall measures can be helpful to draw a representation of the users and their habits. In the manuscript, we used the overall SNS use in two ways: the first to see if the overall utilization has increased during the quarantine measures (page 10, lines 225-230), the second to see if this overall utilization is associated with the motivations to use social network sites (page 10, lines 230-234). We also discussed these preliminary results in the discussion section: “**First of all, participants’ increase in the use of all SNSs during the quarantine measures strengthens the need to explore the relation between SNSs and well-being.**” (page 15, lines 347-348), and “**Rather, it seems that users’ motivations are more indicative: Twitter and TikTok use during quarantine were not related with social relationships, contrary to Facebook and Instagram. But while TikTok use was only related to entertainment, Twitter use was also related to some purposive values which are considered as a subtype of social support [i.e., informational support, 32].**” (pages 16-17, lines 385-392). We hope this is a sufficient clarification.

- concerning your data, I understand your limitations, and that's fine, but you should state more clearly in the abstract and the introduction sections that this is a preliminary work.

We thank the reviewer and agree with his/her suggestion. To be clearer about our study’s scope and limitations, we added more information in the abstract section: “**Future research is required to go beyond methodological and statistical limitations and allow generalization.**” (page 2, lines 41-42). Similarly, we have also updated the introduction section: “**The present study will contribute to this research question, through a short literature review and an empirical study. More research will be required to provide a clear understanding of how SNSs impact our well-being.**” (page 3, lines 57-59). We hope we have correctly addressed the reviewer’s concern.

# Reviewer #3:

I would like to thank the authors for such a quick and thorough revision.

From my point of view all of the suggested modification have been implemented except for item 1).

The descriptive statistics and correlation table following the link

https://osf.io/s5mjx/?view_only=b852a4a3eb884b8bb11b83256bee0161

shed little light.

I would still recommend trying to visualize the exploratory data analysis (EDA) in terms of histograms and KDE plots of PDFs. See, for example, https://seaborn.pydata.org/generated/seaborn.pairplot.html

One could separate the group of actively using social networks per network by its median into two and plot PDFs for positive affect, negative affect etc questions, or directly use x-y scatter plots with with KDE.

I would encourage to perform such EDA for all available variables, including age, gender etc

With EDA plots the reader would be prepared and actually expect the result. The benefits of EDA include dataset consistency check and motivation of the model: is the input dataset biased and what conclusion should we expect from the model?

We thank the reviewer for pushing us to develop our analyses further. We agree with all his/her suggestions and made the necessary changes: “**An exploratory analysis of the data is available in OSF at: https://osf.io/s5mjx/?view_only=b852a4a3eb884b8bb11b83256bee0161 (for blind peer review, this link leads to the general project on OSF, but not directly to the statistical summary document. You can find it in the "Data Appendix" folder.).**” (page 10, lines 222-225). First, we gave some basic summary statistics of all the study variables (mean, median, standard deviation, minimum, maximum). Then, we used histograms and KDE plots to give a visual representation of them. Finally, based on the median split method, we separated the usage into two groups – high and low – and plotted histograms according to the study variables. By doing this exploratory data analysis, we learnt a lot about our data, and we thank the reviewer for that. For example, we saw that the passive and active usages seem, even if the literature considers them as separate constructs, slightly associated. We therefore discussed it as a limitation in the discussion section: **Second, to avoid demotivating respondents, we have limited the questionnaire’s length. Consequently, passive and active SNS usages have been measured with one item. Although they are considered as separated constructs in the literature [13], we may suspect that the use of single items has increased their association. Future studies should therefore assess specific activities on each SNS.**” (page 17, lines 405-409). We hope having correctly addressed the reviewer’s concern.
