# Supplementary material for: Don’t put all social network sites in one basket: Facebook, Instagram, Twitter, TikTok, and their relations with well-being during the COVID-19 pandemic
Source: PLoS One. 2021 Mar 11;16(3):e0248384. doi: 10.1371/journal.pone.0248384 (PMC7951844; doi:10.1371/journal.pone.0248384)
Supplement: Attachment — Submitted filename: Response to Reviewers.docx [file pone.0248384.s003.docx]

**Response to the Editor and the Reviewers**

We are re-submitting in *PLOS One* our paper "Don’t put all social network sites in one basket: Facebook, Instagram, Twitter, TikTok, and their relations with well-being during the COVID-19 pandemic.".

We thank again the editor and the reviewers for the time they spent reviewing our manuscript.

Below are detailed responses to all the reviewer’s comments. The latter are shown in black and our responses in blue.

Sincerely,

The authors of paper PONE-D-20-27590.

**Page**

[Reviewer #1	2](#_Toc61000487)

[Reviewer #2:	3](#_Toc61000488)

[Reviewer #3:	4](#_Toc61000489)

# Reviewer #1

Reviewer #1: I appreciate the data exploration analysis that the authors have included in the current revision.

As the authors have satisfactorily addressed the comments, I consider that the manuscript can be accepted for publication in PLOS ONE.

We thank the reviewer for the time he again devoted to our paper, but also for considering that it can be accepted for publication in PLOS One.

# Reviewer #2:

# (No Response)

# Reviewer #3:

The exploratory data analysis was nominally performed, however, it is completely detached from the rest the analysis (and not very informative).

Usually it serves the purpose of motivating further analysis using more advanced techniques.

For instance, in lines 266-268,"Age was associated with satisfaction ... with life negative affect ... and positive affect"

The significance and the signs of effects should be manifest in EDA.

I believe any reader would appreciate an announcement of strong correlation in the prelude and a following confirmation by a stronger method.

We thank again the reviewer for his/her advice. We made the necessary changes: “**An exploratory analysis of the data is available in OSF at: https://osf.io/s5mjx/?view_only=b852a4a3eb884b8bb11b83256bee0161 (for blind peer review, this link leads to the general project on OSF, but not directly to the statistical summary document. You can find it in the "Data Appendix" folder.).**” (page 10, lines 222-225). We have kept the summary statistics and KDE plots, but we also computed a correlation matrix and displayed scatter plots. The readers will be able to see what the data tell us before structural equation modeling. For example, the direction of the relation between age and well-being is simpler to anticipate. We hope we have, this time, correctly addressed the reviewer’s concern.
